# Donation After Circulatory Death: A New Frontier

**DOI:** 10.1007/s11886-022-01798-y

**Published:** 2022-10-22

**Authors:** Yashutosh Joshi, Jeanette Villanueva, Ling Gao, Bridget Hwang, Christine Zhao, Aoife Doyle, Jianxin Wu, Paul Jansz, Peter Macdonald

**Affiliations:** 1 Heart & Lung Transplantation Unit, St. Vincent’s Hospital Sydney, 390 Victoria St, Darlinghurst, NSW 2010 Australia; 2 Victor Chang Cardiac Research Institute, Darlinghurst, NSW Australia; 3 Faculty of Medicine, St. Vincent’s Clinical School, UNSW, Randwick, NSW Australia

**Keywords:** Donation after circulatory death, Heart transplantation, Heart failure, Organ preservation, Normothermic machine perfusion, Normothermic regional perfusion

## Abstract

**Purpose of Review:**

To highlight the current global experience with DCD heart transplantation and explore the evolution of, and compare preservation strategies; examine early clinical outcomes, and discuss the growing use of DCD donors as a new frontier in heart transplantation.

**Recent Findings:**

The two strategies of DCD heart preservation include NMP using the OCS Heart and TA-NRP followed by either: NMP or CSS. Better understanding the limits of cold ischaemia following TA-NRP will aid in distant procurement. Asystolic warm ischaemia plays an important role in determining immediate post-operative graft function and potential need for mechanical support. Large volume DCD heart transplant units show no difference in survival between DCD and DBD donor heart transplants.

**Summary:**

In a previously non-utilised source of donor hearts, often viewed as an “unknown frontier” in heart transplantation, DCD hearts are a suitable alternative to brain-dead donor hearts and are likely to remain a permanent part of the heart transplantation landscape. Global uptake is currently increasing, and as understanding of preservation strategies and tolerable ischaemic times improve, utilisation of DCD hearts will continue to grow.

## Introduction

Heart failure represents a significant disease burden on healthcare systems worldwide—patients with end-stage heart failure (ESHF), refractory to maximal medical therapy, face frequent hospitalisation, and high morbidity and mortality [1]. In this patient population, heart transplantation remains the optimal treatment but remains limited by donor organ availability [2, 3]. Annually, it is estimated that 6000 heart transplants are being performed globally, with the number of heart transplants performed demonstrating an overall increasing trend over time [3–5]. However, recipient listings and waitlists have also grown, creating an increasing gap between demand and supply of donor hearts [6]. Arguably contributing to this growing waitlist is an expanding population of patients with advanced heart failure, an increasing acceptance of older and more medically complex recipients, and improvements in and increased utilisation of mechanical support therapies allowing for more patients to be bridged to transplantation [3–5].

Given these trends, increasing the number of donor hearts sourced is of great interest and importance in order for supply to meet demand. With the majority of heart transplants traditionally being performed from donors after brain death (DBD), donors from a donation after circulatory death (DCD) pathway are emerging as a key alternative source of donor hearts [7••, 8••].

With heart transplantation from DCD donors still in its relative infancy, in this review, we aim to explore the evolution of preservation strategies that have allowed an increasing uptake of DCD donors as a source of donor hearts, examine early global clinical outcomes, and highlight the growing role and use of DCD donors as a new frontier in heart transplantation.

## The Long Journey of DCD Donor Hearts

Inspired by surgical and preservation techniques pioneered by Dr. Norman Shumway and his team [9], the first clinical orthotopic heart transplant performed by Dr. Christian Bernard in 1967 was in fact from a 25-year-old DCD donor [10]. In this landmark operation, the donor heart was retrieved following a 5-min stand-down after cessation of electrocardiographic activity; cardiopulmonary bypass was then established and the heart ultimately selectively perfused and cooled to 16 °C after which point the donor heart was excised, placed in 10 °C Ringer’s lactate, and transported to the adjacent theatre where it was immediately reperfused and subsequently implanted [10].

Although the recipient ultimately succumbed to pneumonia and acute rejection after 18 days, the foundation had been laid for future heart transplants to follow [11]. Subsequent to this, the number of heart transplantations began to increase globally, aided in particular by the 1968 Harvard Medical School Committee statement defining brain death [12]. This led to a global adoption of BD donors in whom, as death had been declared based on neurological, rather than circulatory criteria, an assessment of the beating heart was possible after death and prior to organ procurement [4, 11].

Despite the world’s first heart transplant being derived from a DCD donor, over the next four decades, donor hearts were almost entirely retrieved from BD donors and were preserved in cold-static storage (CSS) [11]. The reluctance in using DCD donors can be attributed to the donation pathway necessitating a cessation of circulation involving variable periods of warm ischaemia prior to organ procurement [13]. The heart is sensitive to warm ischaemia, and in a DCD donor is also subjected to a period of severe pulmonary vasoconstriction during withdrawal of life support (WLS) resulting in an additional insult to the right ventricle [14, 15]. Consequently, at the time of procurement, the DCD heart is in a distended, non-beating state. In contrast to BD donors where the beating heart can be assessed in situ prior to organ procurement, concerns surrounding the potential impact of WLS on the myocardial viability prevented the use of DCD donor hearts, particularly as there was an inability at the time to assess cardiac viability following cessation of circulation [13, 16].

Over time however, uptake of DCD donation for other organs increased with acceptable post-transplant outcomes. Transplantation of kidneys retrieved from DCD donors showed no difference in survival compared to those retrieved from BD donors [17] and gradually acceptance of other organs from DCD donors such as the liver, pancreas, and lungs followed suit [13]. Heart transplantation from DCD donors re-emerged in the paediatric setting. Boucek et al. from Denver Children’s Hospital reported the first three successful cases of paediatric heart transplants from DCD donors [18•]. Noteworthy (and controversial) features of these transplants were the measures taken to minimise the warm ischaemic insult to the donor heart during WLS and subsequent procurement [16]. These included co-location of donor and recipient in adjacent operating theatres, antemortem cannulation of the donor to allow rapid administration of preservation solution after death, and a shortened “stand-off” time of 75 s in the 2nd and 3rd cases. In all 3 cases, CSS was used to preserve the heart while it was transferred to the adjacent operating room. Subsequently, the International Society of Heart and Lung Transplantation (ISHLT) reported a total of 21 paediatric DCD heart transplants (HT) between 2005 and 2014 [19]. The ISHLT reported paediatric DCD-HT recipients to have a significantly higher requirement for post-operative mechanical support and 1-year mortality compared to DBD-HT recipients (though it should be noted that a higher proportion of the DCD-HT recipient cohort were on pre-operative mechanical circulatory support suggesting higher pre-operative risk) [19]. Location of donor and recipient and the mode of preservation of the DCD hearts were not reported but it is likely that in the majority if not all cases a similar protocol to that reported by Boucek et al was utilised [18•]. While this paediatric experience provided “proof of principle” that DCD hearts could be recovered and successfully transplanted, in order for DCD heart transplantation to significantly address the worldwide organ shortage in adult heart transplantation, it was clear that there was a need to develop procurement and preservation strategies that allowed for not only an assessment of the viability of the DCD donor heart following procurement and prior to implantation, but also a means to allow DCD donor hearts to be distantly procured [18•, 19].

## Breakthroughs in DCD Donor Heart Preservation Strategies

With growing global interest into DCD donors as a means to increase the donor pool, pre-clinical research conducted by our unit at the Victor Chang Cardiac Research institute in Sydney laid the groundwork for our unit to commence a clinical DCD heart transplant program at St. Vincent’s Hospital in 2014. In a porcine DCD model, hearts subjected to 30 min of total warm ischaemia from the time of withdrawal of life support (WLS) followed by administration of a supplemented Celsior cardioplegic solution were able to demonstrate functional recovery during normothermic perfusion on an ex situ working heart apparatus (after 1 h of Langendorff perfusion) [20]. This study prompted consideration into the role of ex situ perfusion in the setting of DCD heart preservation.

The introduction of the TransMedics (Andover, MA, USA) Organ Care System (OCS) Heart normothermic machine perfusion (NMP) device as a means of reperfusing donor hearts with normothermic, oxygenated blood in a resting (or Langendorff) fashion played a vital role in the translation of our pre-clinical findings into a clinical DCD heart transplant program [21]. The Proceed II [22] trial had demonstrated that NMP with the OCS Heart was non-inferior to cold-static storage in the setting of BDD-HT recipients.

The use of NMP allowed, for the first time, an assessment of donor heart viability ex situ—the lack of which had previously inhibited the uptake of DCD donor hearts. Pre-clinical work by our unit demonstrated NMP with the OCS Heart to be superior to CSS in a porcine DCD transplant model [21]. On the basis of these findings, in 2014, our unit performed the world’s first series of distantly procured DCD donor heart transplants [23]; we have since performed 77 DCD heart transplants in Australia to date [24].

Following our initial experience, the uptake of DCD donor hearts began to occur globally. In addition to using the OCS Heart, the Royal Papworth Hospital in the UK also began to retrieve DCD hearts using thoraco-abdominal machine perfusion (TA-NRP) [8••, 25]. TA-NRP emerged as an alternative to NMP for assessing DCD donor heart viability, and its uptake has seen DCD programs appear more recently in Belgium [26••], Spain [27••], and the USA [28••, 29••, 30••].

## Withdrawal of Life Support

DCD donation processes involve the withdrawal of life support (WLS). In DCD donors where the heart is being retrieved, WLS has generally occurred in a controlled environment such as in an intensive care unit or anaesthetic bay (Maastricht Category III donors). Occasionally (usually at the request of the donor family), retrieval of the donor heart and other organs has occurred after controlled WLS in brain-dead donors (Maastricht Category IV donors) [7••, 31]. Once WLS has occurred, retrieval teams then await donor progression to circulatory arrest—this usually must occur within a certain time frame or the organ is rejected. The maximum times that teams are willing to wait following the withdrawal of life support varies between countries, as does the designated stand-off period and definitions of functional warm ischaemic times. Table 1 illustrates known published thresholds for retrieval teams following the withdrawal of life supports.

**Table 1:** Global published adult DCD preservation protocols and retrieval timings

**Country/centre**	**DCD heart preservation protocol**	**Withdrawal of life support time frames**	**Mandatory stand-off times following asystole**
Australia (St. Vincent’s Hospital, Sydney) [7••, 24, 39••]	NMP	From WLS to SBP (SBP) < 90 mmHg: 90 min From SBP < 90 mmHg until asystole: approx. 30 min	5 min (prior to 2021, 3–5 min)
UK (Royal Papworth Hospital) [8••]	NMP TA-NRP followed by NMP/CSS	From WLS to asystole: 120 min From SBP < 50 mmHg to asystole: 30 min	5 min
Spain (University Hospital Marqués de Valdecilla-IDIVA/University Hospital Virgen de la Arrixaca/University Hospital Puerta de Hierro) [27••]	TA-NRP followed by CSS	Following WLS once SBP < 60 mmHg until asystole: 30 min	5 min
Belgium (University Hospital of Liege) [26••]	TA-NRP followed by CSS	-	Circulatory arrest declared once femoral arterial pressure non-pulsatile and SBP < 30 mmHg, followed by 5 min stand-off time
United States of America - Vanderbilt University Medical Center, Nashville [28••]	TA-NRP followed by CSS	Following WLS once SpO2 < 70% OR SBP < 50 mmHg until asystole: 35 min	Up to 5 min (minimum time in published series was 2 min)
- Mayo Clinic, Jacksonville [29••]	NMP	Following WLS once SBP < 50 mmHg until asystole: 27 min	5 min
- Lagone Health, NYU, New York [30••]	TA-NRP followed by CSS	From WLS to asystole: 180 min	5 min

Adapted from: Scheuer SE, et al. J Heart Lung Transplant. 2021 Sep;40 (9):882–889, with permission from Elsevier) [49]

In Australia, death determined by circulatory criteria is defined as irreversible cessation of the circulation. Based on this definition, re-establishment of the circulation by normothermic regional perfusion is not permitted after death has been determined by DCD pathway. For this reason, we developed a direct procurement protocol (DPP) for retrieving hearts from DCD donors. Our retrieval team waits up to 90 min following the WLS for the donor systolic blood pressure (SBP) to fall below 90 mmHg [7••]. Once SBP is < 90 mmHg, asystole must then occur within 30 min or the heart is declined. Should the donor progress to asystole, a further 5-min “stand-off” time is observed in order to ensure there has been no auto-resuscitation of the heart [7••]. In Australia, the process of WLS mainly occurs in ICU, with family often present. In some hospitals, WLS occurs in the anaesthetic bay of the operating theatre. After the “stand-off” period, which has recently been standardised to 5 min in all states and territories, the donor is taken to the operating room and is prepped and draped for the organ procurement procedure to rapidly begin as described by Chew et al. [7••].

Total warm ischaemic time (WIT) is the duration between WLS and administration of cardioplegia at the time of organ procurement. Different units have varying definitions for the functional warm ischaemic time (fWIT). In our unit, this is duration between SBP < 90 mmHg and cardioplegia. We refer to the period of time from asystole to the administration of cardioplegia as the asystolic warm ischaemic time (aWIT). Several measures have been adopted to minimise the fWIT. One measure that has been implemented in Spain, Belgium, and certain USA units has been to undertake WLS with the donor prepped and draped in the operating room prior to WLS negating the accumulation of WIT during transport [27••, 30••]. Another more controversial measure has been to shorten the “stand-off” time. During the first reported series of paediatric DCD donors, the “stand-off” time was controversially lowered from 3 to 1.25 min [18•]; currently, “stand-off” times range from 2 to 20 min in different countries with 5 min being the “stand-off” time most commonly used (see Table 1).

## From Donor to the Recipient: Normothermic Machine Perfusion and Thoraco-abdominal Normothermic Regional Perfusion

Two main strategies currently assist in assessing viability of DCD donor hearts: normothermic machine perfusion (utilising the OCS Heart) and normothermic thoraco-abdominal regional perfusion.

### Normothermic Machine Perfusion

NMP is the method of reperfusion in Australia for DCD donor hearts as TA-NRP is not allowed. In regions where TA-NRP is not possible, NMP is also used in the UK [8••]. The heart is procured using a DPP that has been described by Chew et al. [7••]. To briefly surmise, a sternotomy is performed and the right atrium is quickly cannulated in order to drain 1.2–1.5 L of donor blood necessary for reperfusion on the OCS Heart. This process may take between 1 and 1.5 min and it is important to be mindful of concomitant abdominal retrieving teams during this process as it is necessary for the abdominal organ preservation solution to be with-held until blood collection is complete. Following this, an aortic cross clamp is placed and cardioplegia administered followed by routine cardiectomy. Once excised, the heart is then placed in a bowl of ice and cold saline where it is prepared for cannulation onto the OCS Heart where it is reperfused with warm, oxygenated donor blood. Blood flows into the aorta in a retrograde fashion, perfusing the coronary arteries with coronary effluent being ejected by the right ventricle—note the left ventricle is vented and is in an empty “resting” state. NMP allows for an assessment of RV contractility; furthermore, throughout normothermic machine perfusion, serial arterial blood gas samples are able to be measured for correction of electrolytes, pH, and lactate levels. In order to be considered viable, lactate extraction must be demonstrated (venous lactate lower than arterial lactate) with an overall reduction in lactate levels over time [7••].

In high-risk donors in whom pre-retrieval coronary angiography may not be possible in certain locations either due to policy or availability of hospital resources, the OCS Heart can safely facilitate ex situ coronary angiography upon return to the recipient hospital in order to exclude coronary disease [32]. Haemodynamic parameters during NMP that are observed include mean aortic pressure and coronary artery flow; we aim for this to be between 65 and 90 mmHg and 650 and 850 mL/min respectively [22, 33, 34]. Abnormally high mean aortic pressures during NMP can potentially be caused by coronary artery disease and could warrant further investigations such as angiography [35].

The true meaning of lactate profiles in the setting of NMP is yet to reach a consensus; initially thought to be a predictor of graft failure, the initial experience with the OCS Heart had clinicians aiming for a lactate level of < 5 mmol/L to determine if hearts were suitable for transplant [22, 36]. More recent reports, however, suggest that the lactate level at the end of machine perfusion may not be as sensitive a marker for graft failure as originally thought [37]. In the UK experience, when examining 51 DCD heart transplants requiring the OCS Heart, there was no association found between arterial lactate profiles during NMP, rising arterial lactate profiles, or arterial lactate profiles > 5 mmol/L and mechanical circulatory support post-transplant [38]. In our own St. Vincent’s experience, we have moved away from requiring lactate levels to be < 5 mmol/L and instead have determined DCD hearts to be viable if extraction was demonstrated and if the overall lactate profile trend was downward.

To date in Australia, 77 DCD hearts have been successfully transplanted using the OCS Heart [24]. The approach to viability that we employed and would recommend considers the following criteria: (i.) a satisfactory visual assessment of RV contractility, (ii.) favourable lactate profiles, and (iii.) acceptable haemodynamic parameters obtained on the OCS Heart. We believe our favourable outcomes justify this approach. We have found no difference in mortality between BD-HT and DCD-HT recipients since our DCD program began [24, 39••]. Furthermore, the overall rate of extracorporeal membrane oxygenation (ECMO) support for primary graft dysfunction (PGD) in DCD-HT recipients is 15.6%—this rate is 7% when examining the last 54 DCD-HTs conducted since our initial experience was published [7••, 24]. This compares favourably to the Papworth experience with DPP for DCD-HT recipients, who have reported an ECMO rate of 18% post-transplant [8••].

### Thoraco-abdominal Normothermic Regional Perfusion

TA-NRP is an alternative to NMP as a means for assessing DCD donor heart viability through an in situ assessment of DCD donor hearts. It was initially pioneered in donor DCD hearts destined for transplantation by the Royal Papworth Hospital group as a means of providing a functional and structural assessment of the donor heart without having to rely on OCS Heart lactate profiles, which were a point of conjecture [8••, 40]. The general principal of TA-NRP relies on central or peripheral cannulation in order to establish a bypass circuit where donor blood is oxygenated and re-circulated, *after* the donor has been declared dead [8••, 26••, 27••, 40]. By clamping the aortic arch vessels, recommencement of oxygenated cerebral circulation is prevented. If the heart is weaned off bypass and should subsequent evaluation of cardiac function prove satisfactory (via Swan-Ganz and trans-oesophageal echocardiogram assessment), the heart can be considered suitable for transplant and is then excised in the usual fashion and transported to the donor using either NMP or traditional CSS [8••, 26••, 27••, 40].

The surgical approach to TA-NRP varies between countries in regard to the timing of interventions during the donation process. As antemortem cannulation and heparinisation is not permitted in the UK, once the mandatory 5-min “stand-off” period is observed, the Papworth technique employs a central cannulation strategy following median sternotomy in order to establish bypass with heparin being administered directly into the heart [8••, 40]. The published work from US centres: The New York University, Langone Health [30••]; Vanderbilt University Medical Center (Nashville, TN) [28••]; and Mayo Clinic (Jacksonville) [29••] report similar approaches and have outlined their techniques. In other protocols such as in Spain [27••], Belgium [26••], and the initial paediatric experience in the US [18•], the donor is cannulated peripherally via a femoral approach and antemortem heparin is administered.

The Papworth group in the UK has the largest reported experience to date with TA-NRP with 22 DCD hearts being retrieved following TA-NRP assessment of organ viability [8••]. Three of these hearts were then subjected to CSS with donor/recipient being co-located and were excluded from final analysis (patients in whom extended cold ischaemic time was expected due to pre-existing LVADs were excluded from CSS); 19 of these hearts were subsequently perfused and transported on the OCS Heart [8••]. In geographical locations where TA-NRP retrieval was not possible, DCD hearts were retrieved utilising a DPP (similar to the St. Vincent’s protocol) with subsequent reperfusion and assessment on the OCS Heart [8••]; no significant differences were found in mortality or rates of mechanical support between this group and DCD hearts assessed utilising TA-NRP. Similar to the St. Vincent’s experience, no differences in mortality were found between overall DCD-HT recipients and DBD-HT recipients [8••].

The Vanderbilt University Medical Center, Nashville, TN, has published to date the largest known cohort of DCD donor hearts assessed through TA-NRP and then preserved with CSS [28••]. Results remain in the early stage however are promising with no recipients suffering from immediate severe post-operative PGD (sPGD) and no ECMO requirement for PGD. Mean TA-NRP time was 56 ± 8 min and mean cold ischaemic time was 145 ± 27 min [28••]. The multi-centre Spanish experience [27••] is another program in its early stage of development with 4 reported DCD-HT recipients following TA-NRP/CSS. Cold ischaemic times (CIT) ranged between 55 and 80 min, and all 4 patients survived to discharge and there were no cases of post-transplant mechanical support. Tchana-Sato et al. [26••] similarly published promising early results from their first 2 DCD-HT recipients following TA-NRP/CSS; both recipients were co-located in an adjacent operating room to the donor.

Going forward, as more centres adopt TA-NRP/CSS and potentially foray into distant procurement, the role extended CITs play on eventual graft function will be of particular interest in determining the optimal transport strategy between CSS and NMP. The Belgium experience of the first paediatric heart transplant from a distantly procured DCD donor using TA-NRP/CSS reports a cold ischaemic time of 117 min [41] and represents a step forward from the first paediatric DCD heart transplantation where donor and recipient were co-located [18•]. In adults, mean CITs reported by Hoffman et al. [28••] provide an early insight into DCD hearts preserved in CSS following TA-NRP not currently being adversely affected; DCD retrievals in this experience however were limited to donors < 35 years of age. With contemporary programs accepting older donors, the impact of CIT in older donors in the setting of distant procurement is yet to be evaluated; these early results however make it likely that these boundaries will be pushed.

## Ischaemic Times and Mechanical Support

In the St. Vincent’s initial experience with DCD heart transplantation, we noted an ECMO rate of 35% for PGD in the first 23 patients who were transplanted with DCD hearts following OCS Heart NMP [7••]. During this initial experience, it was evident to us that the aWIT played a key role in determining DCD donor heart graft function as DCD-HT recipients who required ECMO had a significantly higher aWIT (15 ± 3 min compared to 12 ± 2 min in recipients not requiring ECMO, *p* = 0.002). For the purposes of our protocol, we define “cold ischaemic time” as the time taken from the delivery of cardioplegia during donor heart procurement until the time of reperfusion on the OCS Heart. Cold ischaemic time was not associated with an increased risk of ECMO.

Since that initial series, our program has grown and we have been able to learn from our experiences along the way. The addition of tirofiban to our blood collection protocol during the DPP in response to module filter clotting issues has resulted in the subsequent absence of module filter clotting and is an example of one of the tweaks successfully made during our close to 9-year experience with DCD heart retrieval [42]. Furthermore, as aforementioned, experiences with potential high-risk donors have allowed for the development of novel techniques to allow for ex vivo coronary angiography to contribute to donor heart assessment when required [32].

Whilst our initial ECMO rate was high, our ongoing use of the OCS Heart has enabled ongoing familiarisation with DPP techniques and an appreciation of the need to minimise aWIT. The ECMO rate in the subsequent 54 DCD heart transplants that have occurred since the initial series is now at 7% [24]. Despite our cold ischaemic time being significantly higher in this more contemporary cohort, this did not significantly impact ECMO rates and neither did significantly longer fWIT times. Different countries have different definitions of fWIT times; however in considering the importance of ischaemic times in DCD donor hearts, our experience lead us to believe that the aWIT arguably plays the most important role in predicting graft dysfunction [24, 39••]. Recent findings in serial endomyocardial biopsies of human hearts not destined for transplant support this notion in demonstrating that markers of cardiac cell dysfunction are intimately associated with periods of prolonged (> 10 min) circulatory arrest [43].

The future approach to DCD hearts should be to consider ways to minimise aWIT in particular, or to develop strategies of allograft preservation aimed at mitigating potential cellular damage that may occur during aWIT.

## Clinical Impact

The implementation of a DCD heart transplantation program has been shown to have a tangible impact on heart transplant wait lists. Messer et al. [8••] report an overall increase in heart transplant activity by 48%. In Australia, our 9-year experience with DCD heart transplantation has resulted in a 26% increase in heart transplantation; furthermore, since 2020, 30% of all heart transplants have been from DCD donors [24, 39••]. More than half of our DCD-HT recipients required a redo sternotomy at the time of transplant (due to previous cardiac surgery, including durable ventricular assist device implantation) reflecting the complexity of patients that DCD-HTs have been able to relieve from the transplant wait list [24, 39••].

Starting a DCD clinical heart transplantation program requires significant resources, coordination, and training. Utilising normothermic machine perfusion involves logistical and financial consideration into the transportation and constant monitoring of the OCS Heart. In contrast, TA-NRP requires the initiation of cardiopulmonary bypass and the management of a heart–lung bypass machine and oxygenator.

Adopting either TA-NRP or NMP is a decision made based on the specific needs of the transplant unit, local laws, and the geographical areas serviced. In Australia for example, due to the size of the country, a DPP followed by NMP with the OCS Heart is advantageous in facilitating distant procurement to interstate and rural retrieval locations [7••, 23].

## Future Directions

The administration of antemortem heparin in DCD donors is an ethically challenging subject, with no clear international consensus [25, 44]. There is currently variable clinical practice with regard to the use of antemortem heparin in DCD organ retrieval. Certain countries practising TA-NRP allow for pre-withdrawal peripheral cannulation and administration of heparin [27••]. In Australia, the permissibility of antemortem heparin varies between ICUs and states. This variability is due in part to the complex ethics surrounding DCD transplantation and to the dearth of scientific evidence to support a practice of antemortem heparin administration [25, 44]. The role of antemortem heparin needs to be better understood. Pre-clinical studies performed by our unit on a rodent DCD model have shown antemortem heparin to be associated with improved recovery of donor hearts following a DCD protocol [45].

Following withdrawal of life support, the physiological milieu is one of profound acidosis; data from our DCD retrieval experience demonstrates mean pre-withdrawal pH to be 7.41 (± 0.06), and this drops to a mean pH of 7.10 (± 0.23) following asystole [46••]. This acidosis is likely a result of reduced oxygenation leading to lactic acidosis generated from anaerobic metabolism, which contributes to both intracellular and extracellular acidosis [46••].

Acid Sensing Ion Channel 1a (ASIC1a) plays key roles in mediating cellular injury in response to acidosis [46••, 47, 48]. A proton-gated cation channel, acidosis triggers the recruitment of serine/threonine kinase receptor interaction protein 1 (RIP1) to ASIC1a, where the phosphorylation of RIP1 results in downstream signaling resulting in necroptosis. Inhibition of ASIC1a has also been shown to protect rodent heats from ischaemic reperfusion injury following an ischaemic insult [46••]. Pre-clinical studies in our laboratory have shown an improvement in cardiac function and recovery in a rodent DCD model when Hi1a, an inhibitor of ASIC1a derived from funnel-web spider venom, is used as a supplement to cardioplegia [46••, 48]. These results warrant further exploration into the cardioprotective role Hi1a may play in clinical DCD heart transplants.

## Conclusion

Data from two of the longest and largest DCD heart transplantation programs in Australia and the UK have demonstrated that survival from DCD heart transplantation is equivalent to traditional DBD donors. DCD programs are now emerging across Europe and the USA with early success. It is now established that the uptake of DCD donor hearts has the impact of improving overall heart transplantation numbers and can legitimately contribute to the reduction of global transplant wait lists. The decision to choose between NMP and TA-NRP retrieval strategies is multifactorial and location dependent. Ongoing research and clinical experience into pushing the boundaries of preservation strategies will provide the impetus for the ongoing evolution of DCD heart transplantation.

## Abbreviations

ASIC1a: Acid Sensing Ion Channel 1a
aWIT: Asystolic warm ischaemic time
CSS: Cold-static storage
DBD: Donation after brain death
DCD: Donation after circulatory death
DPP: Direct procurement protocol
ECMO: Extracorporeal membrane oxygenation
ESHF: End-stage heart failure
fWIT: Functional warm ischaemic time
HT: Heart transplantation
ISHLT: International Society for Heart and Lung Transplantation
NMP: Normothermic machine perfusion
OCS: Organ care system
PGD: Primary graft dysfunction
RIP1: Kinase receptor interaction protein 1 kinase
TA-NRP: Thoraco-abdominal normothermic regional perfusion
WIT: Warm ischaemic time
WLS: Withdrawal of life support
